# From sacred education to street exploitation: the Almajiri Crisis in Nigeria as a nexus of public health failures, legal paralysis, and global security risks

**DOI:** 10.1186/s13010-025-00191-1

**Published:** 2025-09-30

**Authors:** Saifullahi Idris Umar, Sadiq Muhammad Maaji

**Affiliations:** 1 SIHS, Department of Public Health, Symbiosis International University, Pune, India; 2 Department of Public Health, Ministry of Health, Kano, Fagge, Kano State, Nigeria; 3 SIHS, Department of Public Health, Symbiosis International University, Pune, India

**Keywords:** Almajiri, Tsangaya, Public health, Legal reform, Insecurity, Child neglect, Mental health, Nigeria, Islamic education, Global security, SDGs

## Abstract

The Almajiri system, historically rooted in Northern Nigeria's precolonial Islamic scholarship, has devolved into a complex humanitarian crisis. Once a revered educational tradition, the system is now associated with street begging, child neglect, disease vulnerability, and radicalization risks. This paper critically examines the historical evolution and current realities of the Almajiri system, highlighting how colonial disruption, post-colonial policy failures, and socio-economic inequalities have transformed it into a breeding ground for child vulnerability. The analysis reveals a range of adverse health outcomes, including malnutrition, communicable and non-communicable diseases, and untreated mental health conditions. The paper also underscores the system’s link to broader legal and security concerns, including violations of child rights, susceptibility to recruitment by extremist groups, and potential global health risks such as antimicrobial resistance. Despite numerous reform efforts, entrenched cultural norms, governance deficits, and poor implementation continue to hinder sustainable solutions. Addressing the Almajiri crisis requires culturally sensitive reforms rooted in historical understanding, public health imperatives, legal accountability, and multisectoral collaboration. Without urgent and sustained intervention, the Almajiri system will remain a major barrier to national development and global health security.

## Introduction

West Africa is known for its fame in education and trade. Historically, the region was one of the destinations for many scholars from different parts of the world. Islam reached the western part of African provinces in the eighth century and reached northern Nigeria in the ninth century through trans-Sahara trade [127],NOUN, 2021).

Islam and Islamic education flourished in the 13th to fourteenth centuries, with support from the Muslim rulers of West Africa. Mansa Musa established the Masjid of Sankore, which was converted into Sankore Madarasah (center for learning), which had the capacity to house 25,000 scholars and had the largest Library in West Africa, with over 400,000 manuscripts. [140, 172]. The establishment of similar institutions in northern Nigeria was done by the Mais (Kings) of Kanem-Bornu from the 13th to the eighteenth century. Muhammad El-Kanemi (renowned Muslim scholar and ruler of Kanem in the eighteenth century established many schools for traditional Islamic education and provided funding and invited competent Islamic scholars for the same (FCT EMIS, n.d.; [87],NOUN, 2021). The migration of scholars to Kanem-Borno in search of knowledge became apparent during the reign of El-Kanemi, marking the beginning of “Almuhajirun,” an Arabic word for migrants – first used to described the companion of Prophet Muhammad (PBUH), who migrated from mecca to Medina-Ethiopia (Abyssinia) to escape prosecution by Pagan Arabs (Last [114]), used in West-Africa to describe a person who leave the comfort of their home in search of religious knowledge (Singular – Almuhajir, Plural – Almuhajirun) [68],Last [114].

The patronage of traditional Islamic education by the kings of Kanem-Borno over centuries attracted scholars and Ulama from different parts of West Africa (Dr. K. A. Y. Gazali & Mohammed, 2023a; NOUN, 2021). The established schools were called Sangaya in Kanuri and Tsangaya in Hausa [106]. Initially these schools were accessed by children from the neighborhoods from the comfort of their homes [32]. Almajiri is a Hausa translation of almuhajirun, referring to a person who travels in search of knowledge. However, over the decades this word receives other meanings, taking any of these forms: “(a) A child who engages in some form of labor to earn a living. (b) Any person, irrespective of gender, who begs for assistance on the streets or from house to house as a result of some deformity or disability. (c) Children between the ages of 7 and 15 years who attend informal religious school who equally roam about with the purpose of getting assistance or alms.” [68]Tsangaya schools are now being called by names such as makarantar allo,^1^ makarantar toka,^2^ makarantar muhammadiyya,^3^ and makarantar soro^4^ [94].

The Tsangaya system was well regulated with a clear line of communication and an oversight committee. Teacher vetting happens annually during the general gathering of SAMNO MALLAWAMBE; books written by each teacher are submitted for authentication and approved curriculum is given to each tsangaya school (Dr. K. A. Y. Gazali & Mohammed, 2023b). The Dan Fodio Jihad of 1804 also supported the regulation of tsangaya through the establishment of the Inspectorate of Qur’anic Literacy (IQL)—a regulatory body for Islamic education [81],Nmah, Patrick Enoch, 2017). The British invasion of 1904 disrupted the oversight structure and defunded the schools. This vacuum allowed underqualified teachers—Gardawa^5^—to exploit the system for personal gain, turning it into one associated with child neglect [28]. The well-regulated system produced many renowned scholars and businessmen, such as Alahassan Dantata [81, 94], while the disrupted system exploited by Gardawa produced the likes of abubakar Shekau [146].

Today almajiri is characterized by street begging, parental abandonment, child abuse, labor, and systemic neglect. [126, 169, 208]. There are about 13 million of these children roaming the streets of northern Nigeria [44]. These children are mostly from impoverished or polygamous families, often having many children that they cannot provide for. According to Edinyang et al. [68], the system enables parents to abdicate responsibility while Mallams^6^ rely on child begging as a source of income.

The worsening economic situation and rising food insecurity in Nigeria exacerbate these challenges. Almajiri children struggle to have two meals daily [182], and routine immunization campaigns exclude them, which puts them at particular risk for many vaccine-preventable diseases. Additionally, their poor living conditions make them susceptible to communicable diseases such as malaria, typhoid, hepatitis, cholera, COVID-19, and schistosomiasis, as well as mental health disorders [10, 132, 193]. Their social isolation places them at risk of developing social conduct disorder, potentially leading them to theft, phone snatching, vandalism, poor social relationships, sexual abuse, drug abuse, and delinquency later in life, all of which undermine societal productivity [74, 173, 175].

In the context of rising insecurity in Northern Nigeria, Almajiri children have become the prime target for recruitment by violent non-state actors [3, 14, 164]. Additionally, the lack of water sanitation and hygiene (WASH) in tsangaya schools forces these children to practice open defecation, contributing to disease outbreaks like cholera and typhoid, especially during the rainy season (J. M. [39, 128]). This situation worsens the global rise in neglected tropical diseases (NTD) and antimicrobial resistance (AMR) [46, 69],*WASH and Health Working Together*, 2020).

Although several studies have focused on Almajiri children, to the best of our knowledge, no research has comprehensively examined the nexus of systemic failure, public health consequences, community ripple effects, and global health implications. This study seeks to bridge this gap by exploring the historical evolution of the Almajiri system, the emergence of its current practices, and their multifaceted implications."

## The evolution of the Almajiri system: from sacred to exploited

### Traditional Islamic education in Northern Nigeria: the precolonial era

Northern Nigeria had a millennium-old system of Islamic education before the British colonial invasion. Islam and Islamic education reached Nigeria concurrently because it’s a tradition of Islam that, without knowledge, cannot be practiced or even said to have been rooted [110]. In the 8th to ninth century, Islam and Islamic education reached the Kanem-Borno, a strategic location on the Trans-Sahara trade route, connecting West Africa and Central Africa to North Africa and Mecca (Dr. K. A. Y. Gazali, 2014). Kanem-Borno was part of the Bilad-As Sudan (land of the blacks), as posited by the Arab geographers, lying on the shortest, safest, fastest, and busiest trans-Saharan trade route. This strategic location attracted traders from North Africa and other parts of Africa (Dr. K. A. Y. Gazali, 2014; Dr. K. A. Y. Gazali & Mohammed, 2023a; NOUN, 2021). Although Islam reached Nigeria in the ninth century, its practice was not well established until the eleventh century, when Kanem-Borno had its first Muslim ruler (Umme Jilme) (NOUN, 2021).

Mai Umme Jilme of Kanem-Borno was known for his respect for Ulama, scholars, and scholarship. During his reign, he granted Mahram (a certificate of exemption from state services and taxation to the Ulama); this gesture attracted many scholars and Ulama of international reputation to Kanem-Borno (Dr. K. A. Y. Gazali, 2014). The grant of Mahram not only exempted the Ulama and scholars from state services but also granted them honor, security, and freedom to propagate Islam. After the demise of Mai Umme Jilme, his son Dunama I (1097–1150) took after him. Islam and Islamic scholarship in Kanem-Bornu saw a different light during the reign of Dunama II (1221–1259), Who made contact with the North African Muslim countries; by 1257 Kanem embassy was established in Tunis. The relationship between Kanem-Borno and other Muslim countries in North Africa was so good, to the extent that hostels and colleges were built for students of Kanem (Madarasa Al-Rasheed) [18],NOUN, 2021).

The reign of Mai Idris Alooma in Kanem Borno further supported the propagation of Islam. He established schools and mosques similar to those in other Muslim countries and built a hostel in Mecca for the Kanuris from Kanem-Borno [1],K. A. Y. [79],NOUN, 2021). Kanem-Borno saw a decline from the sixteenth century through the nineteenth century.

Sheikh Muhammad Al-Amin El-Kanemi was a product of Al-Azhar University, Egypt, and founder of the Kanem Empire in the eighteenth century, who furthered Islamic education and practice in the region and beyond. Birni Ngazargamo was the capital of the Kanem Empire (FCT EMIS, n.d.). El-Kanemi established a Qur’anic school at Ngala Kanem, now Ngala local government. He established schools, attracted scholars from across the Muslim countries, fended off the Fulani Jihadists through literary dialogue and provided support for Ulama and scholarsrs in Borno [65],FCT EMIS, n.d.; Dr. K. A. Y. Gazali, 2014). Each tsangaya is headed by the Ulama who founded it, and each tsangaya has two levels: the elementary level (supervised by the teacher’s representative) and the advanced level (supervised by the teacher himself) (Dr. K. A. Y. Gazali, 2014).

Tsangaya schools were originally funded by the emirates, and through donations, families were also encouraged to send food to the tsangaya for the sake of Allah. The tsangaya also actively engaged in agriculture to supplement the efforts of the communities (K. A. Y. [79]. The system was well regulated,it had a well-structured governing system with clear lines of communication and leadership. In Kanem-Borno there is an annual gathering of scholars and Ulama called *SAMNO MALLAMAWABE* – an annual Islamic conference where religious matters are discussed and deliberated, newly written books are authenticated and copies sent to Al-Azhar University for further authentication, teachers’ identity, history, knowledge, and credentials and the credentials of their teachers are verified, curricula are developed for use as guidelines in the tsangaya schools, and certification for new Ulama is done (Dr. K. A. Y. Gazali & Mohammed, 2023b).

Similarly, Dan Fodiyo, after his jihad and establishment of the Sokoto Empire, established a regulatory body for educational institutions under the Sokoto Emirate and named it the *Inspectorate of Qur’anic Literacy* (IQL), which regulated the practice of Islamic training and certification of teachers and schools [81],Nmah, Patrick Enoch, 2017). The inspectors document and report their observations to the emirates. Under this supported system of education, the Ulama from Kanem-Borno spread far and wide to spread Islam and religious education as missionaries [17],Dr. K. A. Y. Gazali & Mohammed, 2023a), and their impact was felt across the Hausa land.

To further support the tsangaya system, an entity called “*Gardawa*” was created to provide security for the tsangaya and run some errands for the Ulama. Gardawa were given a mirrored cap to differentiate them from the public; they were also granted some privileges, such as taking things from the market free of cost. Over time, having enjoyed so many privileges, the Gardawa began to rape married women in Birni Ngazargamo [28], the reason why the authority expelled them from Birni Ngazargamo. They dispersed across Borno and set up their tsangaya in an attempt to compete with the Ulama for identity and leadership. Some Gardawa began to claim prophethood, while others became intolerant towards the communities they lived in and began having conflict with the residents [28]. Although there were reports of corrupt practices connected to gardawa before colonial intervention, such tendencies were controlled under the emirate and the rule of traditional authorities. Traditional authorities were treated with almost divine authority, and the words and acts of traditional authorities were unquestioned by traditional subjects [25]. This deep-seated cultural trust facilitated compliance with traditional authority and leadership pragmatics,allowing leadership to supervise religious pedagogues, enforcing ideological conformity, and protecting the welfare functions of the tsangaya structures.

### Traditional Islamic education in Northern Nigeria: the colonial disruption

In 1903 British forces invaded northern Nigeria, setting up their businesses and mining infrastructures. The result of the British invasion into northern Nigeria was the death of many traditional rulers and the deposition of many others, while other rulers were left with empty titles as traditional rulers without the power to preside over the activities of their people (AbdulQadir, 2003). The British colonial masters promised to the residents of northern Nigeria that they would not interfere with their religious or cultural practices, a promise they could not keep. They rejected the education system they met in northern Nigeria and established their own system to serve their purpose [17, 150]. The residents of northern Nigeria felt that the British schools, which were part of their Christian-colonial conquest, would eventually replace their system of belief and identity, making them develop a mixed reaction towards the system [17, 28].

The collapse of the northern traditional system of leadership and the British attempt to subjugate Islamic scholarship and practice in northern Nigeria were viewed by many northerners as the end of time [4]. This perception, coupled with the uprising of Muhammad Ahmad Mahdi in Sudan against the European colonialists, inspired many (local Mahdists) across the Muslim world, including Nigeria, to rise in resistance to the colonial Christian conquest. The Mahdi uprising resulted in widespread armed insurrection and the death of many across northern Nigeria and some parts of southern Nigeria, making the Mahdist uprising short-lived [4, 171]. The British colonialists faced a series of aggressions from many other groups, the deadliest was from Satiru in Sokoto; however, their superior gun power and support from the traditional rulers granted them victory over the Mahdists [4]. The Ulama in northern Nigeria were deliberately forsaken by the British invaders, their role as Qadhi (judges) relinquished, and the application of Shari’ah Law (judgment in accordance with the Qur’an and other Islamic principles) was restricted. The Ulama ignored the restriction and continued to judge in accordance with Shari’ah. Shari’ah was later categorized under the customary laws directly supervised by the British colonizers; some Ulama still refused to comply with the British way [4].

Even though the British invaders succeeded in collapsing the traditional leadership structure in northern Nigeria, they could not get the residents to trust their system of education. Later, they changed strategy by integrating some religious books into their curriculum. However, this system of education did not seem to enjoy any funding benefit or attention, further weakening the traditional Islamic education of northern Nigeria [17]. To further suppress Islamic education and leadership, the teachers employed to teach Arabic and Qur’an in the British-operated schools were grossly underpaid, and other Islamic sciences like Shari’ah, Tawhid, and Fiqh were abandoned. Job opportunities and certification were given to those trained on the principles of the British education system [4, 17, 57].

These unfortunate occurrences in northern Nigeria led to the development of hatred towards Western education and the use of the derogatory term “Boko”^7^ to describe it further stigmatized the system among the northerners [28]. Parents in northern Nigeria chose to send their children to individual Ulama who would travel to the outskirts of villages away from the control of British invaders [57, 107], setting up their tsangaya across many villages, receiving support from the families and communities to run the systems. This heavy responsibility of taking care of the children’s needs falls solely on the shoulders of the Ulama, a situation that could not last long due to worsening support from the community and the parents, making the Ulama and their students engage in menial jobs to sustain themselves (AbdulQadir, 2003) setting the foundation of the almajiri system as seen today.

### Traditional Islamic education in Northern Nigeria: the post-colonial distortion

The collapse of the traditional Islamic leadership of northern Nigeria and the emergence of modern Islamic school (Islamiyya) in 1942 [28], provided alternative for families to send their children to [171], leading to withdrawal of many children from the tsangaya system further worsening the support structure, the weekly stipend (Kudin Sati)^8^ paid by the families of children in tsangaya schools could no longer support the burden placed on the teachers, so students were made to pay daily stipend; a situation that made the students to go about taking menial jobs in the markets, hotels, homes and other public places to make get the required amount [171], making the students to lose both their educational requirement and material needs.

This situation was later exploited by the Gardawa (underqualified, self-accredited teachers who lack knowledge and understanding of the Holy Qur’an and other Islamic sciences), who made use of the loopholes for personal gain. These semi-literate teachers see the current almajiri system as a means of survival (AbdulQadir, 2003). Later, the Gardawa began to send their almajiri to beg, claiming that begging is better than stealing [28]. Additionally, the Gardawa exploiting this unsupervised system spread across the nation. Their teaching method and practice are far from the teachings of Islam. A notable figure among the Gardawa was Marwa Maitatsine (meaning Marwa who curses), Whose nature of preaching and intolerance resulted in his deportation from Kano in 1962 during the reign of Emir of Kano Muhammadu Sanusi I. However, Sanusi was deposed in 1963, giving Maitatsine the opportunity to get back to Kano, which later led to him becoming so strong with many Gardawa under him [28]. In 1980 he led the Maitatsine movement, which resulted in the death of many in Kano State; his remnants dispersed [28, 154]. Lastly, the emergence of Boko Haram insurgents headed by radical Gardawa-like Muhammada Yusuf and Abubakar Shekau [91, 146] is also a consequence of the distorted beliefs of the almajiri system.

The radicalization of people is largely due to the knowledge gap and the rising number of economically and socially disadvantaged youth. Faith without knowledge is dangerous [49],this, coupled with the rising youth bulge syndrome [24], worsens the dangers around these practices. The distorted belief that almajiri should live a lowly life, coupled with the health and physical dangers they are exposed to, makes them particularly vulnerable to mental health illnesses, abuse, and recruitment by violent non-state actors or forming their own criminal groups, as seen today with Yandaba in Kano [163], Boko Haram in the Northeast, and bandits in the Northwest [146, 164, 173]. The nature of their living conditions and reliance on begging to cover distances daily make the almajiri children unskilled andnd therefore unemployable [19],Jeremiah, Ph.D & Vincent Chi, 2025), and their exposure to violence and abuse (Daily [59, 169] makes them learn violence problem-solving skills instead of non-violence approaches.

The system today has degenerated to the extent that almajiri children are mostly sleeping on the streets, haggardly dressed, hungry, barefoot, and destitute without parental support (Jimoh [105]). They are described as parasites, without any value to the society, while many studies link them to criminalities [91, 92, 146]. The nature of the system exposes them to many health risks, including substance abuse disorders and physical injuries [54],Jimoh [105, 132]. Many attempts at improving the system by the government have been labeled as an attack on Islam by the teachers (Jimoh [105]), while others view the almajiri system as a sacred system that does not need reform [54]. Many studies indicated parental wish to abandon their responsibilities or to lessen burden as the reason for sending children to the almajiri schools [54, 67]. The recent widespread abuse of almajiri children by Mallam and others [23],Daily [59, 169] calls for a review of the system.

## Public health failures and the Almajiri crisis

### Food security and nutrition

Almajiri children as socially isolated cohort are seen in every corner and street begging for food and money to make ends meet (Dalhat, 2016; [208]). This practice of survival on begging particularly exposes the almajiri children to food insecurity, which is linked to many health risks [43], including undernutrition [182],psycological distress [10, 102], and increase risks of spread of infectious diseases [200] Climate change, regional conflict, urbanization, and worsening poverty have pushed many families into food insecurity in Nigeria (World Food Programme, 2024a), with over 86% of adults ever experiencing food insecurity and 44% experiencing severe food insecurity [153]. Almajiri, a child relying on leftover food from the neighborhood for survival, is even more at risk of food insecurity.

Studies conducted among almajiri indicated that 66.7% eat once and 33.7% eat twice daily, with the prevalence of malnutrition among almajiri children in Zamfara reported to be 71.9%, 70.9%, and 68.5% of those from Tsafe, Maradun, Kaura-Namoda being malnourished, and approximately 64% of those from Talata-mafara and Anka [167, 182]. Worsening poverty and food insecurity in northern Nigeria are threatening families (World Food Programme, 2024b),over 106 million families are projected to be living in extreme poverty [112], and the recent worsening of malnutrition in the region [103] might affect the source of almajiri livelihood. The practice among the almajiri community that almajiri should provide food for the mallam and his family on a daily basis further threatens the security of the children (UNICEF, 2020b). Further worsening their vulnerability is the imposed weekly levy (Kudin Sati), working on Mallam’s farm or fetching water for the Mallam [28, 85]. Additionally, malnutrition has been a global concern with an estimated direct and indirect cost of $3.5 trillion globally or $500 per individual, and the direct cost for managing malnutrition is estimated at $1 to $2 trillion globally [80]. Recently, due to funding shortfalls, the World Food Programme (WFP) will be forced to suspend food and nutrition assistance for 2 million people affected by crises in April 2025, and urgently requires US$ 620 million to ensure continued support to crisis-affected people across Sahel and Nigeria for over 6 month duration. Without immediate action the Western Africa is expected to be hit by extreme hunger, with 52.7 million to be affected between June and August 2025, out of which 33.1 million will be from Northern Nigeria. Further worsening food security [204]. Malnutrition is also associated with poor academic performance and lost productivity [80, 144].

## Poor living conditions and communicable diseases

The Living conditions of almajiri children are particularly concerning and call for urgent attention from the concerned authorities, parents, mallams, and the communities. It was reported that 100% of almajiri children in Sokoto State sleep in an overcrowded environment, while about 50.6%, 43.3%, and 49.4% of almajiri children in Zamfara were strongly believed to be sleeping in streets, uncompleted buildings, and motors, respectively [167, 183]. 90% of the almajiri schools in Sokoto have no standard hygiene practices, and 67% of almajiri schools in Sokoto have no access to water sources [128]. Although 67% of the almajiri children wash their hands after using the toilet, none of them wash their hands before eating food, and about 67% walk barefoot [167]. These practices of almajiri children are known predisposing factors for communicable diseases and neglected tropical diseases (NTD) such as soil-transmitted helminths like hookworms. However, the provision of adequate water, sanitation, hygiene, and protective footwear receives less attention. Food insecurity, malnutrition, and poor living environments among the almajiri children expose them to communicable and non-communicable diseases [167, 200], and eating all kinds of leftover food gotten through scavenging (Jimoh [105] or given from the neighborhood could expose them to acute gastroenteritis, typhoid perforation, cholera, and other related health risks.

Good water sanitation and hygiene (WASH) have been strongly linked to improved health through the prevention of waterborne diseases. Improved WASH is also linked to the prevention and elimination of neglected tropical diseases (*WASH and Health Working Together*, 2020). Nigeria has been battling with waterborne diseases such as cholera and enteric diseases, with 73% of the burden seen among the poor population. Only 26.5% of the Nigerian population has access to an improved water source, and 22% of the population defecates in the open space. Waterborne diseases result in a high under-5 mortality rate of about 70,000 annually [188]. Access to poor water sanitation and hygiene can lead to the widespread of diseases like polio, typhoid, and cholera,it also makes it difficult to stop the spread of neglected tropical diseases such as schistosomiasis, trachoma, and guinea worm [51]

NTDs are regarded as diseases of the poor due to their prevalence among the poor population, where hygiene is limited [11, 46]. WASH is limited among the almajiri communities due to lack of access to clean water sources and lack of facilities for defecation, worsening the vicious cycle of disease and poverty. Additionally, walking barefoot, especially on soil contaminated with human or animal feces, can increase the risk of contracting certain Neglected Tropical Diseases such as hookworm, strongyloidiasis, and podoconiosis [86, 181],this practice is common among the almajiri children. The prevalence of schistosomiasis is also found to be high among the almajiri children compared to peers attending public school. The prevalence among almajiri children was reported to be 59.6%, compared to 31.5% in public school children [9]. Another study by Yunusa E.U et al.,2016 reported 49.0% prevalence of urinary schistosomiasis among the almajiri participants of the study with reason been majority of participants wash their clothes and other items in river or stream (89.0%), bath with water from river (70.9%), and swim in river or pond (51.5%). [207] this findings were slightly above a pooled prevalence measure for Nigeria which was 34.7% (95% confidence interval [CI]: 31.0, 38.5%) from a systematic review conducted from 1994 to 2015[5]. Intestinal parasitosis prevalence among the almajiri community in Zaria (Kaduna State) and Konduga (Borno State) was reported to be 83.2% and 80.9%, respectively, with prevalence among southern regions (Southwest, Southeast, and South-South) being 28%, 55.2%, and 67.2%, respectively [62, 206]. The overcrowded living conditions, poor hygiene, and lack of shelter are known predisposing factors to malarial infection and severity [53, 64]. Almajiri child who usually sleep outside and are thus exposed to mosquito bites, there by predisposing them to malarial infection, Repeated malaria exposure however, could induce pre-immunity resulting in asymptomatic infection and increased gametocyte carriage among them [35]. The prevalence of malaria among almajiri children in Kano State was reported to be 35.7%, compared to 19.3%, 30.6% and 36.5% in other northern populations [84]Additionally, inadequate waste management and poor sanitation practices allow ARB and resistance genes to persist in the environment, creating a continuous loop of contamination [16].

During the COVID-19 pandemic, state governments repatriated many almajiri children back to their states of origin due to the fear of the spread of the disease [143]. Out of 244 almajiri repatriated back to Kano State, 193 tested positive, which is considerably high [156]. Tuberculosis is known to spread in an overcrowded space,however, the study conducted on almajiri children in Samaru did not yield positive results [131], though this does not mean the case would be the same elsewhere.

Overcrowding, poor water sanitation and hygiene, food insecurity, and social isolation are also noticeable predisposing factors of non-communicable diseases. The most common NCD among the almajiri children is mental health disorders.

## Mental health of Almjiri child

Many countries recognize psychological distress, such as depression and anxiety, as problematic. There is a noticeable increase in the burden of mental health disorders worldwide (World Health Organization, 2013). It’s estimated that more than 50% of the general population will experience psychological distress at a particular point in time during their lifetime [159],therefore, mental health issues are not peculiar to certain predisposed individuals but a major public health problem that has a lasting impact on the affected individuals, their families, social life, and work environment [63, 159]

An almajiri child system is like a boarding school child, except that the almajiri children are not well cared for and have no one to lay their complaint to. Additionally, the almajiri children are rarely visited by their families (UNICEF, 2020b; [208]). Psychological distress is common among all boarding school children, whether almajiri or not [50],however, the almajiri children, being socially isolated and vulnerable to many adversities, are more prone to developing psychological disorders. In a study conducted in Kaduna among 150 almajiri children, 81.3% reported both being sad and lonely, 68% reported being bullied, and 74.7% reported being sad about the nature of the study (Zubairu H. D1, 2024), and 68.67% repeated feelings of being neglected [55]. Abubakar-Abdullateef et al., [10] noticed the prevalence of depression among almajiri children to be 23%, compared to 8.1% of the public school pupils. Additionally, their study found the prevalence of enuresis, PTSD, substance abuse, and Generalized Anxiety Disorder (GAD) to be 20.2%, 9.9%, 5.6%, and 16%, respectively, among Almajiri children compared to 11.1%, 4.0%, 1.0 and 8.1%, respectively, among the public school pupils.

Children like almajiri that are socially isolated and economically challenged are more at risk of developing social conduct disorder, one of the frequent mental disorders seen in children and adolescents [34, 124]. Social conduct disorder is defined as “a repetitive and persistent pattern of behavior in children and adolescents in which the rights of others or basic social rules are violated (Mental Health America, n.d.). The prevalence of conduct disorder varies with country,studies conducted on school-going children in the southern part of Nigeria have shown a prevalence ranging from 9.8% to 15.8%, with higher prevalence seen in adolescents [15, 75]. Most of those who had “conduct disorder” during childhood are repeatedly convicted of crimes such as vandalism, theft, and assault in adolescence, with widespread drug misuse and often alcoholism [71]. According to Mental Health America, conduct disorder is characterized by “

- Aggressive behavior that causes or threatens harm to other people or animals, such as bullying or intimidating others, often initiating physical fights, or being physically cruel to animals.
- Non-aggressive conduct that causes property loss or damage, such as fire-setting or the deliberate destruction of others’ property.
- Deceitfulness or theft, such as breaking into someone’s house or car, or lying or “conning” others.
- Serious rule violations, such as staying out at night when prohibited, running away from home overnight, or often being truant from school.” (Mental Health America, n.d.). Some children engaged in sexual harassment, including rape [26].

Many studies and reports linked the almajiri children to many antisocial activities, including drug abuse, theft, vandalism, sexual harassment, armed conflicts, communal violence (such as Daba in Kano) and regional violence such as Boko Haram and banditry (Katami et al., 2023; [163, 164, 171]). The radicalization of many almajiri children and the emergence of violence by non-state actors could be linked to the development of social conduct disorder during early childhood, perpetuated by their disadvantaged socioeconomic condition. Most of those involved in violence show no remorse even after being apprehended or convicted, which is a typical sign of conduct disorder (Mental Health America, n.d.). About 90% of those diagnosed with conduct disorder progress to becoming repeated juvenile offenders and during adulthood they often develop abnormal lifestyles and engage in violent relationships with others, including partners [162]. The disorder itself is a precipitating factor for multiple other mental disorders and is associated with aggression and violence [117, 129, 130].

Many studies have linked aggression and violence to childhood adversity [22, 165], and this can result in the use of violence, including the use of weapons for conflict resolution. Childhood adversities are drivers to antisocial disorders [74, 165], and antisocial disorders have been a common diagnosis among many criminals, including murder convicts [27, 194]. Antisocial personality disorder, formerly known as psychopathy and characterized by total disregard for the feelings of others, was diagnosed among murder convicts such as Ted Bundy, a necrophile Who was convicted of 30 murders and John Wayne Gacy, Who was convicted of raping and killing 33 people [45]. Many other mental illnesses, such as schizophrenia, post-traumatic stress disorder (PTSD) and depression, are also common. Boko Haram's gruesome killing by throat slitting [20], violent communal clashes in the Kano metropolis (Fadan Daba) and phone snatching using knives can be linked to mental illnesses and drug use [123, 163].

Other mental health disorders such as depression, anxiety, and PTSD noticed among the almajiri children are also found to have a direct and indirect negative impact on employment and the economy. It's estimated that mental health disorders will cost the world $6 trillion by 2030 (excluding anyone outside the paid service), with LMIC bearing 35% of this loss (DINARTE-DIAZ, 2023). Mental health disorders are found to worsen poverty and poverty is found to have a direct link with common mental diseases (CMD), feeding a vicious cycle of mental health and poverty [119]. Other factors associated with mental health that have a direct negative effect on the economy and well-being are food insecurity, work absenteeism and presentism, years Lived with disability and drug abuse blocking progress to SDGs, especially 2 (Zero Hunger) and 3 (Good Health and Well-Being) [42, 43, 47, 102]. Nigeria is facing a gross lack of support resources for psychological distress, with fewer than 200—300 psychiatrists, accounting for 1 psychiatrist to 700,000–1,000,000 populations [149, 184].

Mental health illness, a bad circle of friends, social isolation, lack of parental care, food insecurity, poor access to healthcare and reliance on begging for survival made almajiri prime targets for recruitment by violent non-state actors and targets for abuse (physically or sexually), further worsening internal security and the already fragile economy.

## Impact of Almajiri system on regional instability and global security threat

Over the past decades, Boko Haram's insurgency had grown into a major international security issue, with the emergence of banditry in Nigeria and the neighbouring countries further eroding regional stability. Colonial era disturbances created governance vacuums that over time then gave rise to radical strains of gardawa [28]. From the 1960 s, the hatred for Western politics, education, and culture erupted into highly organised revolts under the leadership of Muhammadu Marwa Maitatsine (1980–1985) throughout Kano, Kaduna, Maiduguri, Gombe, and Yola. Even though the movement was Proscribed, some remnants surfaced in violent activities in Lagos, notably at Bankole Street and Abule-Taylor/Abule-Egba in Agege on 11–12 March and 27 May 1998 [28]. In early 2000 s, Muhammad Yusuf (Founder of Boko Haram Ideology, herein after BH), migrated to Borno from Yobe, where he rose to prominence, and was even part of the Shari’ah implementation committee before turning sharply against western education (*Boko)* and Governance (AFZAL, 2020). Yusuf who was an eloquent speaker had many followers under the movement (Boko Haram – *meaning western education is forbidden),* most of the followers and leaders were former almajiri [91, 146]. This movement was said to have enjoyed political cover by the then state Governor who relied on Yusuf’s influence during the 2003 election, however, this changed in 2009 when the movement had violent confrontation with the police in 2009 that led to the killing of some BH members, making Yusuf to respond with a Jihadist rhetoric, resulting in his execution and killing of over 700 followers (AFZAL, 2020). His death signified a turning moment in the history of the militant group, which then re-emerged under Abubakar Shekau, himself an ex-almajiri [146], Who escalated attacks from targeted killings in Maiduguri to high-intensity bombings, including the 2011 European Union bombing in Abuja and the 2014 kidnapping of those 276 Chibok girls (AFZAL, 2020). The local terror group Boko Haram spread to the other Sahel states when military action against them intensified around 2014 [98],they regrouped in Nigerien territory. Though closely supervised by the Nigerien military, the group was seen as a Nigerian problem until 2016, when they reigned terror on both Nigerian and southeastern Nigerien communities [98]. The Boko Haram insurgent group has been linked with global terror groups like Islamic State (hereinafter IS) or Al-Qaeda [31, 97]. These groups share one thing in common: exploiting the vulnerabilities of socially isolated groups that struggle for identity, resources, or justice.

The vulnerabilities of people living in Nigeria and other conflict-affected areas in the world are being exploited by the non-state actors [97]. Almajiri, a young child having no parental guidance, no community protection, and being hungry and destitute, covering distance daily in search of livelihood, fits perfectly into the category of vulnerable group (Abdullahi, 2020; [118, 158]). These vulnerabilities create a vacuum, one exploited by the regional and international terror groups to radicalize the affected group through coercion or filling the gap for the vulnerable group and providing what they are lacking, from identity to social needs and protection (Balzacq & Settoul, 2022 [97, 152],). In Nigeria, radicalization of vulnerable groups has worsened since the emergence of Gardawa [28]. This tradition of radical Islamism in Nigeria is becoming worse with the current economic and political instability in Nigeria getting worse day by day. The rising security crises in Northern Nigeria have led to the closure of many businesses and abandonment of farmlands, resulting in economic stagnation and worsening food insecurity [101].

Northern Nigeria is having a growing number of youth and unemployment. This situation, coupled with the rising food insecurity and weak economy, creates a situation known as “Youth Bulge syndrome" [24]. The youth bulge syndrome provides opportunity for radicalization of youth and eases their recruitment into violent groups, feeding a vicious cycle of insecurity, poverty, and unemployment. Families reported that sending their children into the almajiri system is safer for them than allowing them to be recruited by the Boko Haram insurgents (UNICEF, 2020a). The ongoing regional instability in northern Nigeria is further tilting the country into a fragile state, which is defined as “a fragile country is currently experiencing fluctuations in economy and politics. It does not possess a strong economy or a well-regulated society. As a result, any small turbulence could bring large impacts on all factors. Moreover, even if the country manages to recover from these disturbances, it can only return to its previous fragile state" [70]. Borno, which was prosperous, had most of its populace, including almajiri, heavily dependent on agriculture; however, the shrinkage of Lake Chad over the past decades resulted in heavy economic losses and displacement of the people to other places in search of livelihood, resulting in competition over limited resources (Li, 2024; [192]). The lake, which was said to be the 11th largest globally, has shrunk by more than 90% from 1963 to 1990, causing massive economic losses to the neighboring countries [100],LI, 2024). It is estimated that by 2050, about 140 million people in Sub-Saharan Africa, Asia, and Europe will be internally displaced persons due to climate change [192],this will worsen the ongoing crises due to the struggle and competition for limited resources available [192],World Bank, 2023). Limited resources, food insecurity, climate change, and the presence of unskilled, unemployed, semi-literate youth produce an enabling environment for non-state actors to thrive.

This protracted regional conflict, coupled with food insecurity, unemployment, poor health infrastructure, and weak governance, is making the efforts of security agencies and government futile and counterproductive (Peace Science Digest, n.d.). The roaming of almajiri children and the increasing number of street beggars are keeping the circle of insecurity, family displacement, and destruction of farms going on for far too long [14, 148, 178], 2024). Borno, Sokoto, and Kano, being the most common destinations of almajiri children, have been battling with some sort of security issues, from Boko Haram in Borno and banditry in the Sokoto region to Daba in Kano. The almajiri system has been the biggest contributor to Boko Haram, banditry, and Daba in Northern Nigeria [91, 163]. Africa’s porous border and weak interstate collaboration between the African countries affected by this regional conflict furthered the thriving of this protracted regional conflict and global security threat [98, 145]. Additionally, Almajiri and other street children are not just unemployed but simply unemployable because they are not trained, so they only resort to violence, theft, burglary, deception, and harassment (sexual or physical) for survival [19].

The popular street gangs of Kano State have been pulling the state down for decades by affecting businesses and restricting movements within the metropolis. The incidences of phone snatching, armed theft, communal clashes resulting in deaths or disability, substance abuse, and arson are very common in the Kano State metropolis [19],A. [56]. This is crippling businesses and costing the state a lot in productivity and revenue. Farmers and herders clash, and Boko Haram's rampant massacre of communities in the northeast and northwest of Nigeria results in the displacement of over 2 million people and the death of many [90]. It also resulted in direct wealth and property loss, including ransom, which was estimated at N2.23 trillion between May 2023 and April 2024 [134]. An additional N9.17 trillion was spent on the purchase of military equipment from 2020 to 2024. Additionally, these activities are leaving a devastating mental health impact on the communities affected, further worsening food security, businesses and employment, and health and healthcare.

Regional instability, climate change, and migration could worsen global health, including AMR, the spread of infectious diseases, and progress toward sustainable developments such as SDG 1 (No poverty), SDG 2 (Zero hunger), SDG 3 (Good health and well-being), SDG 4 (Quality education), SDG 6 (Clean water and sanitation), SDG 10 (Reducing inequality), and SDG 16 (Peace, justice, and strong institutions).

## Global health implications of the Almajiri system

The almajiri system of education and practices has devastating global health implications. Almajiri children are mostly excluded from healthcare budgets, including immunization. Illiteracy and religious beliefs have been cited as the reasons many almajiri children do not seek healthcare from hospitals when sick and reject immunization [198]. The overcrowded living conditions and poor hygienic practices with poor access to clean water expose many almajiri to diseases such as COVID-19, which have the potential to cross borders [30, 143]. The practice of open defecation among the almajiri communities is common due to limited infrastructure for defecation; this practice has been attributed to the global spread of waterborne diseases such as cholera, polio, typhoid, and hepatitis [128, 188]. Additionally, the practice of open defecation in the sewers, roadsides, farms, water sources, and culverts by the almajiri children (T. A. [40, 168]). The prevalence of cholera outbreaks is noticed to be high around Kano [137] and other northern states such as Borno and Sokoto [29, 82]. The almajiri children cannot afford healthcare [93],hence, they can hardly complete a course of antimicrobial when sick, adding to the global antimicrobial resistance.

Antimicrobial resistance is one of the most troubling global health issues that can cross national boundaries and travel to every corner of the earth. This can be fueled by poor water sanitation and hygiene and regional instability [69, 188]. It was estimated that bacterial AMR resulted in 1.27 million deaths and contributed to about 4.95 deaths globally, with monetary costs estimated at $1 trillion by 2050 and GDP losses of $1 trillion to $3.4 trillion by 2030 [199]. Almajiri are also exposed to sexual assault [23, 169], which could predispose the almajiri to sexually transmitted infections (STIs) and worsen the prevalence of these diseases, further burdening the already overburdened healthcare infrastructure. The prevalence of STIs remains high in Nigeria [122, 151], mostly attributed to poor sexual practices and limited knowledge.

Antimicrobial resistance is associated with increased length of hospital stay, disability, or death, and sexually transmitted diseases and other communicable diseases reported in almajiri due to poor hygiene have the potential to increase the burden on healthcare and also worsen food safety and security [199], 2010). The lack of skills, illiteracy, and vulnerability of the almajiri system are a threat to Nigeria’s and West Africa’s economy and food security and a threat to global healthcare. A multisectoral, global collaboration is necessary to tackle the issue of almajiri in Nigeria.

## Legal and policy paralysis

The precolonial legal framework highlighted above regulated the practices around Islamic education before its disruption by the colonial invasion. The colonial invaders tried to disrupt religious practices in northern Nigeria to strengthen their control, which led to the breakdown of the existing legal system and allowed unqualified teachers to take over, changing the once respected education system into the flawed one we see today.

Historically, reforms attempted by both the federal and state governments on the Almajiri system have hardly succeeded, suffering from inadequacies in policy design and implementation. In 1959, the Kano Native Authority issued some directives to warn Qur’anic teachers against the movement of their students to other towns without prior approval, as a strategy to curb child street begging under the guise of Islamic education [2]. In the 1960 s, integrated formal education represented perhaps the first formal attempt at combining Islamic curricula with Western curricula in institutions such as the Nizamiyya Islamic Primary School in Zaria [185]. Kano State passed an edict in 1980 to provide for the registration of Qur’anic schools, which was amended sometime later in 1988 to make provisions for the movement of Almajirai and for the control of their enrollment, partly in response to the Maitatsine crises [111]. In 1986, Sokoto State adopted the “Control of Juvenile Accompanying Qur’anic Malam’s Adoptive Rules” which introduced settlement schemes enabling Islamic and Western education at pupils’ home locations. During the 1980 s and 1990 s, faith-based organisations, including the Islamic Education Trust, the Islamic Trust of Nigeria, the Islamic Foundation, FOMWAN, JIBWIS, and the Da’awah Group of Nigeria, set up some model schools incorporating secular subjects into the teaching of the Qur’an [48],N. M. [58]. At the federal level, the National Policy of Education in 1977 acknowledged Qur’anic education in principle, whilst the Universal Basic Education Scheme of 1999 aimed at universal enrolment, but neither gives effective avenues to integrate the Almajiri system into the formal structure [174].

The 2013 almajiri reform by the Federal Government of Nigeria [176], though a commendable effort, failed due to certain factors. The team assigned to integrate almajiri schools into the regular formal system contained high-profile individuals who had limited knowledge of the psychology of change (Barnes, n.d.). While the almajiri system as practiced today might have outlived its usefulness, reforming it would require a technical and adaptive approach. Limited understanding of the psychology of parents, teachers (mallams), and the students (almajiri) has been one of the notified challenges the implementation team faced (Baban’umma, 2018). When dealing with cultural ties top-down approach might not always be the required approach [115]. Dealing with the almajiri system would require a thorough understanding of the system and the past resentments the system holds on the contemporary formal system. In most cases, representatives from a heavily reliant bureaucratic approach might not have the understanding of cultural differences across communities, so they might not be able to reason with local communities. Similarly, the representatives assigned to integrate the almajiri system into the formal education system had limited understanding of cultural relativism [138],therefore, they were only able to see the material benefits attached to integrating the almajiri system into the formal system (Baban’umma, 2018).

Additionally, the National Commission for Almajiri and Out-of-School Children Act 2023 [21], which was signed into law at the national level, has been trying to reform the system with not much progress. Despite past failures at reforming or abolishing the system, there are still mixed feelings about the almajiri system, with some people suggesting the system be abolished while many others suggest the system be reformed (DAMAGUM, 2024). The Almajiri system as practiced today violates many national and international laws; however, due to poor governance and implementation strategies of past reform efforts, the system seems to be getting even worse and getting out of hand. Recently, reports of abuse of almajiri children by their mallams and community opportunists have been increasing [23],Daily [59, 169].

Constitutionally minded, one may observe that the Almajiri phenomenon actually contravenes basic rights guaranteed in Chapter IV of the Constitution of the Federal Republic of Nigeria [73] [73]. These include the right to life (Sect. 33), endangered by the hazardous conditions created by street life,the right to dignity (Sect. 34), violated by coercion into begging and other degrading forms of treatment; and the right against discrimination (Sect. 42), which is equally violated by the systemic exclusion of the Almajiris in education, health, and other areas.

Being a member of the United Nations and the African Union (AU), Nigeria has ratified and domesticated major instruments safeguarding child rights, including the UN Convention on the Rights of the Child (CRC), the Universal Declaration of Human Rights (UDHR), the ILO conventions, and the African Charter on the Rights and Welfare of the Child (ACRWC). The CRC provides by Article 19 that children shall be protected from all forms of physical or mental violence, injury or abuse, neglect or negligent treatment, maltreatment, or exploitation — a right frequently denied to almajiri children, Who are subjected to violence and exploitation on the streets. Article 24 guarantees the right to the highest attainable standard of health compromised by poor nutrition, lack of medical attention, and social exposure to communicable diseases. Article 28 guarantees free and compulsory primary education, which almajiri children rarely attain as they are given mostly informal education that is without recognized qualification. Article 32 prohibits "economic exploitation and hazardous work", as seen in the prevalent situation of forced begging on streets. Article 34 prohibits all forms of sexual exploitation and abuse, with the risk being exacerbated under conditions of unsupervised living (UNICEF, n.d.). The UDHR also states Article 25, entitled "The Right to an Adequate Standard of Living", subsumes, "the right to adequate food, clothing and housing and, Healthcare" and Article 26—The Right to Education, (UNITED NATIONS, n.d.). According to ILO standards, Convention No. 138 establishes minimum age for employment (15 for labour and 18 for hazardous work) to help ensure that children and young people's education is not compromised, and Convention No. 182 bans the worst forms of child labour, which covers any occupation that negatively affects the health, safety or morals of children and young people — begging and street survival work fall in this category (Internationales Arbeitsamt, 2014). Moreover, the ACRWC specified these rights at a regional level: Article 11 states that governments will provide free and compulsory basic education; Article 14 includes access to health services; Article 15 bans child labour; Article 16 provides protection against abuse and torture; Article 19 guarantees parental care; Article 20 lays out parental responsibility; Articles 21–22 bans harmful cultural practices and protects children from armed conflict (African [12]).

A significant precedent in this context is the decision of the African Committee of Experts on the Rights and Welfare of the Child in Centre for Human Rights (University of Pretoria) & La Rencontre Africaine pour la Défense des Droits de l’Homme v Senegal (Communication No. 003/Com/001/2012) (AFRICAN Union, 2014). In this case, the Committee held Senegal liable for violating several provisions of the ACRWC in that: it had not exercised due diligence to prevent the systematic forced begging of talibés in the Quranic schools (ACERWC, 2015). The decision stated that the state incurs responsibility for a further negligence for the acts of a non-state actor when it fails to ensure due diligence to prevent rights violations. Senegal was ordered to prohibit forced begging, to reintegrate children with their families, and to incorporate and integrate Quranic schools into the formal education system. The Almajiri system in Nigeria reflects the same issues described in this decision—child begging, lack of education, and child abuse. This makes the Senegal case very persuasive for Nigeria, and this decision requires legislative and policy changes to ensure compliance with constitutional guarantees and respect for the regional human rights framework. Beyond breaching these instruments, the current almajiri system undermines progress toward Sustainable Development Goals (SDGs) 1, 2, 3, 4, 6, 8, 10, 16, and 17 (UNDP, n.d.).

Although the Child Rights Act 2003 has not been domesticated across the Northern states, the practice could be seen to violate many sections of the Act, such as Sect. 11 (dignity of child), Sect. 13 (health and health services), Sect. 15 (education), Sect. 19 (responsibilities of child and parents), Sect. 26 (use in criminal activities), Sect. 30 (labor, including begging for alms), and Sect. 31 (sexual abuse and exploitation) (National Legislative Bodies/National Authorities, 2003). The National Child Rights Protection met with resistance and non-domestication from the northern states due to its nationally centric nature [155]. Some of the sections contradict the moral beliefs of the northern states, so the northern states failed to accept and domesticate them.

Many attempts were made by Hisbah (state religious police operatives) to regulate the almajiri practice in Kano State, yet they failed. Some efforts made included attempts to stop late-night roaming by almajiri children [41] and repatriation of the almajiri children to their respective states (Muntari & Agency Report, 2024; [147]), with recent efforts involving rehabilitating the almajiri before repatriation [147]. However, lack of proper design and implementation strategy, coupled with limited understanding of the system, made all the efforts futile.

## Lesson learned from similar system: failures and successes

The menace of Street children is not peculiar to a particular country or region. Many countries with similar challenges to almajiri children of Nigeria have implemented reforms in response to Child neglect, informal religious education abuse, and street exploitation. Examining these systems would provide an insight to developing a culturally sensitive and context specific reform agenda for Northern Nigeria.

In Senegal, the *talibes* are like the almajiri children in Nigeria, being forced into street begging and occasionally being physically and sexually abused by their teachers [89, 157], many attempts by the government to reform the system including the recent ones were viewed as an attack on the Daraas (religious boarding schools in Senegal) by the teachers. Recent government effort which engaged the religious scholars were successfully implemented through phased implementation approach [89, 120].

In India, similar top-down approaches were attempted with minimal successes. However, community led, vocational focused integrative approach by civil society organizations and NGOs have seen some successes. Some of those community level approached are Salaam Balaak Trust (SBT), Bal Mitra Mandal (BMM) and Child-friendly Local Governance. These approached saved thousands of street children and transform them into national assests [108, 121, 161].

In Bangladesh, the government partnered with NGO to provide a multisectoral approach to save its over 1.5 million street children. Save the Children report revealed how community level child inclusive governance and decision making transform the children into community support group. The government and NGO support the children through training and resources provision [170].

In Pakistan, Madarasah reform attempt by the government which aimed at registering and regulating the madarasah was strongly resisted by the scholars in fear of loss of autonomy and politicization. However, progress was made when incentives were involved, such as direct funding and vocational supports. [96]. Similarly, in Indonesia, the Pondok pesantren (Islamic boarding schools) also underwent series of reforms from the thirteenth century to date similar to that of Nigeria. Several attempt at reforming the system was met with resistance from the Muslims communities in Indonesia. Recent successes in reforming the system were after the government recognize the system and fund it at National and local government level and engage the scholars and communities in decision making, thus promoting community ownership and governance [113, 142, 166].

Across these case studies, several lessons emerged. First, community ownership and religious leader’s engagement is crucial to sustainability and legitimacy. Second, legal and policy should be paired with support and possibly incentives not just monitoring and punishment. Third intersectoral collaboration linking health, education, social welfare and child protection is critical for tackling the aforementioned child vulnerabilities. Lastly, reforms that respect cultural norms while integrating children into the formal system proved to be more successful.

## Way forward: multisectoral approach

The degeneration of the millennium-old system of traditional Islamic education in northern Nigeria has become too obvious to be neglected. Although an attempt was made by the federal and state governments to tackle this distorted practice, without a clear understanding and holistic approach, the situation will only get worse. While change is necessary and inevitable, it will always meet resistance and sabotage from those benefiting from the status quo or for other reasons (West, n.d.). The Almajiri system is like any other system; an attempt to change it by reforming it or abolishing it will be met with resistance and sabotage by those benefiting from the system.

The Kano State Qur’anic and Islamiyya Schools Board has been at the forefront of regulating Qur’anic and Islamiyya schools; however, the almajiri schools remain invisible to government policies. To propose a way forward for the almajiri system, we need to have a thorough understanding of the system and the reason previous reform attempts failed. In this document we have seen the historical background of the almajiri system (from a sacred and well-regulated system to a neglected and exploitative system of child neglect, abuse, rejection, and parental abandonment), the health and community implications, the economic consequences, the regional and global security threat, and the legal and policy paralysis.

Most of the tsangaya schools have bitter feelings towards the Western education system, stemming from how British colonial masters used Christian emissaries to forcefully bring their Western ideology to the northern states after killing their traditional rulers and renowned scholars (AbdulQadir, 2003; [17]). The treatment received by the Islamic scholars after the colonial disruption [17] further worsened the resentment of northern Muslims towards the Western education system. The use of the derogatory term “Boko” to label the western education system stigmatizes it among the northern communities [28, 136]. To date, there is a rhyming song among the northern communities about Boko saying those who attend the western education system neither study nor pray [209], all in an attempt to dissuade people from attending the western schools. Additionally, almajiri children are being raised by the product of the same system, generation after generation; they are being indoctrinated into radicalism and extremism and taught to believe that Western education is sinful, making them have bitter feelings towards Muslims pursuing Western education [6, 88, 179]. Additionally, almajiri having limited understanding of Islamic sciences, including poor knowledge of the Qur’an, is exposed to having poor religious tolerance toward other Muslims (AbdulQadir, 2003; [28]). This almajiri community needs to be desensitized to these beliefs.

The notable reasons for resisting change among people are misunderstanding the meaning of change, lack of skills, lack of confidence, fear of the unknown, emotional attachment to the past, poor communication, lack of consultation, seeing change as something that will not last long, breaking the routine, changing the status quo, past resentment, and loss of control and authority [52, 83, 180]. Similarly, almajiri teachers heavily reliant on almajiri children begging for alms to support them and annual subsistence farming [54], p. 17,[85],UNICEF, 2020b; [209] would resist any attempt at revitalizing the system. Additionally, the almajiri system has been a practice among impoverished families, so it is mostly a result of abject poverty [68, 88, 209]. The Almajiri system provides an alternative for families to abdicate their responsibilities [67, 209]. So both the parents and the almajiri teacher benefit from the system, which is why they perceive any attempt at regulating the system as a threat to their source of livelihood. Since they are not skilled at anything else, they would do everything possible to protect their only means of survival. Similarly, elder almajiri would do everything possible to protect the system, since they are next in line of succession. Young almajiri are the only people wanting to exit the system, and many succeeded in escaping [179],UNICEF, 2020a).

## Recommendations

The federal government should understand that protection of culture and economic development of individual states should be the responsibility of individual states. Therefore, instead of a national-level reform strategy, it should be a state-level reform, since each state has its culture and challenges. The state and federal governments should collaborate, and the reform by state should be handled at the state level with support from the federal government. If the state does not see reason to reform its environs, no amount of effort from the federal government would produce any positive result.

The Tsangaya system is part of the Islamic culture of Northern Nigeria; however, the current system of begging and child exploitation has no religious background. The current practice needs regulation; the dignity of these young, promising children should be protected by the government, the community, and the parents while pursuing their Qur’anic education. The institutionalized neglect and parental abandonment should be regulated while the tsangaya system remains in existence.

While the tsangaya teachers have the right to run schools and admit children, the government has the responsibility to protect the children and public security and maintain order. Therefore, an oversight committee like that of the Inspectorate of Qur’anic Literacy (IQL) and SAMNO MALLAMAWABE should be created and integrated into the government infrastructure to preside over the activities of the Tsangaya system. This would ensure order and adherence to laws and ethics in teaching and learning, protecting the children and the community while boosting economic activities.

Age limits should be set for children that can be sent away from home to far-away Tsangaya. While the tradition of sending children away has been centuries old and was a result of limited Tsangaya schools and fear of colonial exploitation and disruption of religious identity, now there are tsangayu in every corner of the northern part of the country. Therefore, if it is true that the parents prefer their children to go to the tsangaya system rather than the formal school, they can do so from the comfort of their homes while enjoying parental guidance and familial care, similar to that of precolonial time [32]. Children below 15 years of age are tender and require parental guidance to be nurtured into responsible individuals (Ilm [95]). Giving too much freedom to children can make them feel neglected and expose them to risks before they have the maturity to handle them, this can lead to them to harmful decision making [195] even when they are raised by their parents,the case of almajiri children is even worse. Those above 15 years can be sent as far as necessary, but even at that, the mallams should be responsible for them. Mallams as legal guardians of almajiri should be responsible for the action of almajiri irrespective of gravity. The parents should send their children to the Mallam with all the necessary provisions they might require to study peacefully. Other necessities Like decent accommodation, good dress, and healthcare should be provided by the parents. Since religious education should be valued more than anything, the one pursuing it should not be left dejected and depressed. Children below 15 years should be back with their families. If the Mallam and families feel they want to keep the children under the care of mallam, they should provide accommodation in the form of boarding school and the children should always be closely supervised by the mallam.

Government should build vocational training centers to convert almajiri from liability to asset. Handcrafts like carpentry, mechanics, shoe-making and shoe shining, other leather crafts like bags, belts, wallets, decorations, etc., electrical/solar installation, tailoring, phone repair, local soap making, and many other local creations that are essential to the development of the state. Teachers should be engaged and provided psycho-social rehabilitation and training on the need for the reform; they should also be offered vocational training and financial support as an alternative to making almajiri children go out to beg for them. Parents of most almajiri are former almajiri; thus, they are mostly unskilled, resorting to sending these vulnerable children away to lessen their responsibilities. Government should engage them in vocational training too. The almajiri children and their families need psychosocial support; a unit should be trained to provide such support after each session of vocational training. Those who have been in the system for quite a long while should be provided counseling and cash of up to N5000 for every almajiri Who attends the counseling session; at least six sessions of counseling should be received by every almajiri. Healthcare should be subsidized for the almajiri and his families; this would reduce the prevalence of diseases and delayed healthcare seeking. Where families own farms, the government should collaborate with them through the Ministry of Agriculture to produce food throughout the year, sharing it with the government at 70:30 (parent: government). This would provide an alternative to the parents.

The lack of regulation and domestication of the Child Protection Act allows the exploitation of children across northern states, with male parents mostly abandoning their responsibilities to the mothers. Where the mothers failed to provide for the children, the male partner would send the child to an almajiri school to beg for survival, while the family have one less mouth to feed.

Verification and certification of Mallams' competencies by the council of Ulama and state Shariah board to ensure that Mallams operating tsangaya are qualified to do so. This certification will follow with registration of each tsangaya; each tsangaya must be endorsed by the district heads and LGA chairman to ensure adherence to laws and ethics and ensure that each Mallam is taking the responsibilities he is supposed to take. Where a tsangaya school violates the set guidelines and the district head fails to report to the local authority, he should bear the consequences on behalf of the tsangaya since the tsangaya was endorsed by him (hence, he would be accountable). Where Mallam is found guilty of breaching the laid guidelines, he should be fined for the first or second offense and be banned from operating the school for repeated offenses. This would set a moral and ethical boundary to child neglect and exploitation and would protect the dignity of the children while preserving community safety. While this bottom-up accountability will be challenging initially, it will be appreciated by the communities and will give the community power to manage and protect their communities. The sense of community ownership is the first step towards community development. Empowering communities to regulate the tsangaya system would take care of issues of cultural relativism, since each community would have a different culture.

Hisbah should be empowered to monitor the implementation of this reform and non-compliance should be handled decisively. Since the lack of regulation and failure of monitoring and evaluation following the colonial disruption of the system is what brought corruption into the system by allowing unqualified, semi-literate Mallams to hijack the system and convert it into what it is today. But Hisbah cannot act without legal backing. A legal infrastructure should be developed and guidelines should be clearly stated so Hisbah would have clear roles and responsibilities to play.

Security agencies should treat almajiri as victims instead of criminals, and where almajiri are engaged in one crime or another, they should be swiftly handled without embarrassing the almajiri. These vulnerable groups had no emotional connection, no parental guidance, and were exposed to different sorts of people on the street, so they learned anything they saw and became what the street made them into. Social media radicalization through films like A DUNIYA, UMAR SANDA, and the like would provide the almajiri with skills like phone-snatching, theft, Daba, kidnapping, and related crimes, which are taught in the movies. If possible, while the state is fragile, movies like that should be temporarily banned, or the content should be modified to support peace rather than criminality. Movie industries should help the government in teaching things that would help the restoration of order in the state and the region. Government should train its media aides in providing security and media literacy to protect people from media radicalization.

Formal education, while important, the parent and almajiri Mallam would see its integration into the tsangaya system as another attempt to subjugate their authority and transfer it to those who have formal literary skills and are more befitting of managerial positions. Therefore, its integration should be systematic and incremental, allowing the parents and the Mallam to see its importance in the long run by slowly showing them its importance and desensitizing them from the idea that formal education, “BOKO,” is a Western ideology that would rob them of their religious identity.

The 2003 child protection act should be reviewed by the state legislature and modified to reflect our cultural practices and norms, then domesticated to protect the state and the children. The set laws would ensure sustainability in child protection and system modification. Clear punishment for non-compliant Mallam should be stated in the legislative laws.

These should be collaborative approaches with the local business owners, philanthropists, federal government, development agencies like the World Bank, WHO, UNICEF, UN, Islamic Development Bank, African Development Bank, African Union, IFAD, IITA, FAO, WFP, USAID, and UKAID; donor agencies like BMGF, Dangote Foundation, BUA Foundation, and UNODC; and NGOs like Save the Children, IRC, MSF, and Plan.

An international conference should be held in one of the one of the hotspots for almajiri children such as Kano [37], sponsored by the donor agencies, to discuss the issue with all the necessary stakeholders, like traditional rulers, historians, Ulama, development agencies, business owners, northern political leaders (including state governors or representatives), renowned tsangaya owners, community elders, philanthropists, and donors. In this conference, the almajiri system would be discussed from its sacred origin to what it is today, then the religious ground on begging and child dignity would be discussed by the Ulama, then public health implications, including the development of a number of mental health issues and their consequences on the society, the community, the nation, and the globe, would be discussed, and then the way forward and strategic planning would be discussed in collaboration with the stakeholders to regulate this century-old distorted practice of exploitation.

Applying this strategy would slowly dissolve the resentments almajiri and their teachers hold towards the contemporary education system and other formalized Qur’anic systems. It would also help transform almajiri from unemployable parasites to assets. The problem has never been about regulating the system but the uncertainty and fear of losing their source of livelihood. A regulated system would help Nigeria a lot economically and morally, but any attempt to make almajiri teachers leave their individual schools would be met with resistance because they see their individual schools as their lifelong investment, which is why you would hardly see two almajiri teachers owning a school together. In this system the mallams struggle for recognition and identity, which is why they recruit as many disciples as possible; the more, the merrier. Regulated systems like Al-Azhar University have been in existence for centuries, producing many renowned religious leaders and still giving scholarships to schools across the Muslim world, including Nigeria (Anyanwu, 2024). Regulating our system and empowering the almajiri teachers with required skills would greatly benefit Nigeria.

## Limitation

This article is primarily a narrative synthesis based on historical and policy documents, and secondary literature. This approach provides a good overview of how the almajiri system evolved, and what the implications have been, however, this has no argument to make for lack of archival documentation and sources taken from interpretations of earlier literature. Further, establishing direct causal links between colonial interventions, policy levers in the post-colonial period, and security outcomes today is challenging when the evidence is often incomplete and mediated by the many socio-political conditions that both precede, and overlap these different periods. Further, in drawing comparisons with systems elsewhere in West Africa, the analysis falls short of its full potential precisely because it is not rooted in primary fieldwork and hence may occlude the localized nuances. Nonetheless, these limitations are not to detract from the importance of the paper in highlighting how health, legal, and security risks interlock through the continuing presence of the almajiri system but are rather pointers toward areas where further empirical and policy-based research is needed.

## Conclusion

The transformation of the Almajiri system from a revered educational tradition into a public health and human security crisis underscores the complex interplay between historical disruption, structural neglect, and governance failure in Nigeria. This study has shown that the current state of the Almajiri system poses significant risks not only to the health and well-being of millions of children but also to national stability and global health security. Chronic exposure to poverty, malnutrition, disease, abuse, and neglect has rendered Almajiri children one of the most vulnerable populations in Nigeria, perpetuating cycles of inequality and contributing to broader societal fragility.

Despite numerous policy declarations and reform attempts, progress has been stifled by cultural resistance, poor political will, and fragmented implementation. The continuation of the status quo represents a collective failure to uphold basic human rights and to protect future generations from structural violence.

Addressing the Almajiri crisis demands urgent, sustained, and multi-sectoral action. This includes integrating health, education, legal protection, and social welfare into a unified response that is culturally sensitive, community-led, and policy-driven. Restoring the dignity and potential of Almajiri children is not only a moral imperative but also a strategic necessity for Nigeria’s development and security**.**

## Data Availability

No datasets were generated or analysed during the current study.

## Abbreviations

IQL: Inspectorate for Qur’anic literacy
IS: Islamic State
SDG: Sustainable development goals
IITA: International Institute of Tropical Agriculture
NTD: Neglected Tropical Diseases
WASH: Water Sanitation and Hygiene
WFP: World Food Programme
PTSD: Post Traumatic Stress Disorder
CMD: Common mental Diseases
AMR: Anti Microbial Resistance
GDP: Gross Domestic Product
STI: Sexually Transmitted Infections
ILO: International Labor Organisation
UN: United Nations
NGO: Non Governmental Organisation
WHO: World Health Organisation
UNICEF: United Nations Children Emergency Funds
UNODC: United Nations Office on Drugs and Crimes
IRC: International Rescue Commission
MSF: Medecins Sans Frontieres
FAO: Food and Agricultural Organisation
IFAD: Intervention Fund for Agricultural Development
MBGF: Bills and Melinda Gate Foundation
FOMWAN: Federation of Muslim Women’s Association in Nigeria
JIBWIS: Jama’atu Izalatul Bid’a Wa’Iqamatus Sunnah
ACRWC: African Charter on the Rights and Welfare of the Child

## Footnotes

1 This is specific to the wooden slate (Allo) the students use. Makaranta is a Hausa term for School.

2 The school is sometimes called makarantar Toka (Ash) – referencing their nature of using wood fire as source of light at Night and Dawn creating file of Ashes.

3 Its religious origin following the teaching of Prophet Muhammad (PBUH).

4 Soro means a veranda, Porch or a covered Platform attached to a house where the students study and sleep.

5 Gardawa practice what is called Gardanci: an ideology far removed from Islamic teachings and from the norms and values of our communities. Different strains of Gardawa have somewhat different beliefs, but one thing is common among them all: a vehement hatred for Western education and Western ideologies.

6 Mallam (plur. Mallamai): A male adult responsible for the care and education of almajirai in a Tsangaya. Also used as a generic honorific in the Hausa language, akin to ‘Mister’ in English. Derives from the Arabic Mu’allim.

7 **Boko:** A derogatory term used in the Hausa Land to stigmatize western education, originated from the postcolonial time, meaning deceit/fake. Aimed to dissuade northerners from sending their children to western schools which were established by Christian emissaries.

8 **Kudin Sati**: Weekly levy, which families to pay to Tsangaya mallams to take care of their children. Mostly paid on Wednesdays. Now imposed on almaijri child, some tsangaya made it daily levy.
